# Effects of pH and Ionic Strength in Calcium on the Stability and Aeration Characteristics of Dairy Emulsion

**DOI:** 10.3390/foods12101976

**Published:** 2023-05-12

**Authors:** Yunna Wang, Xin Cui, Yang Li, Shiran Wang, Guosen Yan, Liebing Zhang, Yan Li

**Affiliations:** 1 Beijing Engineering and Technology Research Centre of Food Additives, School of Food and Health, Beijing Technology and Business University, Beijing 100048, China; 2 Institute of Food Science and Technology, Chinese Academy of Agricultural Sciences, Beijing 100193, China; 3 College of Food Science and Nutritional Engineering, China Agricultural University, Beijing 100083, China

**Keywords:** pH, ionic strength, aerated emulsion, flocculation, colloidal calcium phosphate

## Abstract

The effects of different pH levels and ionic strength in calcium on the stability and aeration characteristics of dairy emulsions were investigated in this study. The results revealed that the stability and aeration characteristics of the emulsion were enhanced as the pH value increased from 6.5 to 7.0 and were optimal within the pH of 6.8~7.0, while the concentration of free calcium ions (Ca^2+^) was 2.94~3.22 mM. With the pH subsequently fixed at 6.8 and 7.0, when the addition of CaCl_2_ was increased to 2.00 mM (free Ca^2+^ strength > 4.11 mM), stability and aeration characteristics reduced significantly, including the flocculation of fat globules, an increase in particle size, and a decrease in the zeta potential and viscosity of the O/W emulsion, all leading to an increase in interfacial protein mass and decreased overrun and foam firmness. Overall, the results indicated that pH changes and CaCl_2_ addition significantly influenced the stability and aeration characteristics of dairy emulsions, by influencing free Ca^2+^ strength, which is an important factor in determining the quality of dairy emulsions.

## 1. Introduction

Whipped cream is a typical aerated dairy emulsion, popularly consumed directly or as an ingredient in confectionery and sweet baked goods [1,2]. This well-known dairy product is a complex oil-in-water (O/W) emulsion that can be transformed into aerated foam via aeration. The stability of whipped cream is determined primarily by the interaction between the protein adsorption layers and the continuous phase’s environmental conditions, such as pH, ionic strength, and concentration of added calcium ions (Ca^2+^) [3].

Notably, the pH condition of O/W emulsions can obviously have an impact on the interfacial composition [4,5,6]. It was previously reported that, when milk was approximately pH 6.5 at the time of heating to above 70 °C, most of the denatured whey proteins were associated with casein micelles, presumably via the formation of disulfide-linked complexes with κ-casein at the surface of the micelles. However, as the pH increased to 6.7, only about 30% of the denatured whey proteins were associated with the casein micelle surface [7]. Allen, Dickinson, and Murray (2006) [8] prepared emulsions with sodium caseinate and groundnut oil and found that the higher the pH of the emulsion during aeration, the higher the overrun. Emulsions with a higher pH are more viscous and envelop more air. During aeration, if the pH of the emulsion is considerably below the isoelectric point of casein, the air is more likely to become enveloped in the system, which increases the stability of the air bubbles. Lactic acid and sodium hydroxide were used to modify the pH of whipping cream to a range of 6.0~7.9 before aeration, and the foam was firmer at the lower pH, softer at pH 7.2, and too soft to whip at pH 7.9.

Colloidal calcium phosphate (CCP), also known as calcium orthophosphate nanoclusters, is typically believed to be present in casein micelles throughout the micelle [9]. The structure of CCP in dairy emulsions can vary depending on its stoichiometry with phosphate (for instance, the Ca/phosphate ratio) and its structure, such as amorphous or crystalline, which affects the way it interacts with the phosphoserine residue of caseins [10]. Reducing the pH causes the progressive dissolution of the CCP so that, at pH < 5.3, virtually all the calcium and inorganic phosphate are found in the soluble phase [11]. Higher concentrations of Ca^2+^ have been shown to reduce the electrostatic charge on the protein-adsorbed layer, resulting in charge shielding and promoting interactions between hydrophobic regions, thereby weakening electrostatic repulsion between bilayers and forming aggregates. Dairy emulsions with higher Ca^2+^ produced stiffer gels with higher viscosity due to increased Ca^2+^-mediated protein–protein interactions in [12]. As the strength of Ca^2+^ increases (0.01–0.05 mol/L), the ζ-potential of O/W emulsions decreases significantly, while viscosity increases and stability decreases. However, the stability increased when a smaller Ca^2+^ dose of 0.10~0.20 mmol/L was added to the emulsion in [13]. Before the protein is adsorbed by fat globules, Ca^2+^ is bound to β-lactoglobulin and α-lactalbumin, resulting in changes in the quaternary structure and tighter binding [14]. It was demonstrated that the addition of small amounts of Ca^2+^ shifts the equilibrium towards the micellar casein phase, lowering the serum casein content induced at low temperatures. This affects all casein species to varying degrees, changing the composition of the casein equilibrium in the aqueous phase. In comparison, adjusting the pH independently of the CaCl_2_ addition had a minor effect on casein concentration and composition in the serum in [15]. The development of stability and rheology in dairy emulsion systems was similarly found by Allen et al. (2006) [8] to be strongly dependent on pH and the concentration of additional Ca^2+^. Ca^2+^ bonded to the surface of fat globules lowers the zeta (ζ) potential, increases the particle size and interfacial protein mass of the emulsion, reduces the electric dipole distance, and destabilizes the emulsion [16]. By reducing the net protein charge and introducing calcium bridges, Ca^2+^ contributes to milk protein gel [17]. During gelation, reduced electrostatic repulsion promotes closer contact and aggregation of casein subunits [18]. As a result of the formation of stabilizing ionic bindings linking the network, more intermolecular network bonds form between adjacent casein monomers or casein subunits, and the gel strength of milk gels increases with increasing calcium content. Soluble calcium alters the calcium equilibrium between serum and micellar colloidal calcium phosphate [19]. The dairy industry is aware of the difficulty posed by the interaction of Ca with dairy proteins and other Ca-binding compounds, which can have an impact on manufacturing processes (such as increased viscosity), instability (such as aggregation, gelation, and phase separation), and acceptance characteristics (such as texture and flavor). Thus, a more profound comprehension of Ca–protein interactions in the dairy emulsion is required to control processing and formulation.

Though both of the effects of pH and CaCl_2_ are well elucidated in protein-stabilized (dairy) emulsions, aerated emulsions such as whipping/whipped cream are quite different from simple emulsions. Because the aerated emulsion is a complex system that consists of not only fat and protein, but also emulsifiers, stabilizers (gel), and salts. Therefore, the whipped cream product needs to achieve a delicate balance between quiescent stability and aeration ease, which are somewhat contradictory attributes in that high levels of quiescent stability require a stable emulsion. In contrast, whipped cream necessitates the aggregation or coalescence of fat globules in the emulsion, which can be easily converted into an aerated system and ensure the firmness of the aerated foam [20]. However, little is known about the effects of pH and free Ca^2+^ on dairy emulsion stability and aeration characteristics.

Hence, anhydrous milk fat (AMF) and milk protein concentrate (MPC) were used as raw materials; an investigation was first conducted to evaluate various parameters specific to the emulsion, such as surface protein mass, ζ-potential, and particle size, in conjunction with aeration characteristics under different pH values and Ca^2+^ additions. The results can provide the necessary theoretical guidance for application in the dairy industry.

## 2. Materials and Methods

### 2.1. Materials

AMF (99.96 wt.%) and milk protein powder 70 (MPC70, 70.31 wt.% protein, 1.33 wt.% fat, and 7.49 wt.% ash) (Fonterra Cooperative Group, Auckland, New Zealand). Glyceryl monostearate (GMS) and microcrystalline cellulose (MCC) (Sigma-Aldrich LLC., St. Louis, MO, USA). To suit the needs of the analysis, all other reagents and solvents were analytical or chromatographic grade.

### 2.2. Emulsion Preparation

All of the emulsions were produced via a standard preparation procedure and a whipping cream recipe with some modifications [21]. The AMF was heated to 80 °C before dissolving the GMS into the liquid fat to form the oil phase. The MPC solution and MCC dispersion were then mixed and homogenized at 80 °C in a two-stage high-pressure APV-1000 homogenizer (Delavan, WI, USA) under 10 MPa for the first stage and 3 MPa for the second stage during the prepared oil phase. After homogenization, the mixtures were batch sterilized at 121 °C for 7 min, immediately cooled at 4 °C, and then aged for 24 h at this temperature before being measured and analyzed. The final emulsion contained 36 wt.% AMF, 0.3 wt.% MCC, and 0.4% GMS. Emulsions were made in triplicate.

After being kept at 4 °C for 24 h, the emulsions underwent aeration. A six-speed electric hand mixer (Hamilton Beach, Richmond, VA, USA) was used to whip the emulsions, first at 950 rpm for 30 s and then at 1100 rpm until the ideal aeration time was reached. The maximum firmness of the aerated emulsion was defined as the time required to reach it, which was also evidenced when the aerated emulsion started to separate from the mixer blades and was no longer in a fluid state [22]. The aeration experiments were repeated twice for each emulsion.

### 2.3. Characterization of the Emulsion

#### 2.3.1. pH Treatment of Emulsion

The initial pH of the emulsions was between 6.9 and 7.0. Hydrochloric acid (HCl) (0.02 M) and sodium hydroxide (NaOH) (0.02 M) were used to adjust the emulsion pH by stirring well in the manner previously described with some modifications [23]. The pH range used for the emulsions was 6.5–7.0.

The pH levels of both treated and untreated emulsions were measured using a digital pH meter (SmartCHEM pH, TPS Australia), which was calibrated with standard buffer solutions of pH 4.0 and 7.0 (Merck, Darmstadt, Germany) before use. All studies were repeated three times on different batches of emulsion.

#### 2.3.2. Ionic Strength

The effects of different CaCl_2_ levels on emulsions were subsequently determined. The pH of emulsions was adjusted to 6.8 and 7.0 using 0.02 M HCL and 0.02 M NaOH before the addition of CaCl_2_ in various final concentrations of 0.25, 0.50, 0.75, 1.00, 1.50, and 2.00 mM. The emulsions were centrifuged at 10,000× *g*, 4 °C, and the supernatants were aspirated with a syringe for later use.

The strengths of Ca^2+^ and Cl^−^ in the emulsions to which CaCl_2_ had been added were determined using a calcium electrode (perfectION™ combined calcium electrode, Seven Compact S220, Mettler Toledo, Singapore), and the resultant potentials were compared with those of the Ca^2+^/Cl^−^ standard buffer solutions, from which the aqueous Ca^2+^ and Cl^−^ strengths were derived [24]. The strengths of PO_4_^3−^ were determined by extraction phosphomolybdenum blue colorimetry [25].

#### 2.3.3. Zeta Potential

The ζ-potential of the variously treated emulsions was measured using a Malvern Zetasizer Nano-ZS90 (Malvern Panalytical, Malvern, UK) at ambient temperature. The emulsions were diluted with distilled water at a ratio of 1:500, after which 1 mL of each diluted sample was placed in a disposable ζ cell (Model DTS 1060C, Malvern Panalytical). Each sample was measured in triplicate.

#### 2.3.4. Particle Size Distribution

Size distributions of the fat globules in the emulsions were determined using a Malvern Mastersizer 3000 (Malvern Panalytical) after a storage period of 24 h at 4 °C. The relative refractive index, dispersed phase adsorption, and continuous phase refractive index were set at 1.52, 0.1, and 1.33, respectively. The emulsions were diluted in recirculating water until they were obscured by 10–20%. Each sample was measured three times.

#### 2.3.5. Creaming Stability

A LUMiSizer (LUM GmbH, Berlin, Germany) was used to evaluate the physical stability of the emulsions. The obtained graph (computer-integrated) revealed the percentage of light absorbance per time interval, also known as the ‘creaming rate’. The multi-sample analytical centrifuge used in this study is equipped with space- and time-resolved extinction profiles (STEPTM) technology (LUM GmbH, Berlin, Germany), which allows for the measurement of transmitted near-infrared light as a function of time and position throughout the entire sample length. The measurements were performed with the following instrument parameters: rotation at 1500 rpm, a time interval of 10 s at 25 °C (after 24 h of storage at 4 °C), and a light factor of 1.00 [25]. Each sample was measured three times.

#### 2.3.6. Protein Mass on Fat Globule Surface

The protein mass on the surface of fat globules in the emulsions was measured according to a method described by A. Ye et al. (2000) [26]. The emulsions were centrifuged separately at 10,000× *g* for 60 min at 4 °C in a TGL-16A centrifuge (Pin Fan Co., Ltd., Hunan, China). The upper layer was carefully removed to measure the difference in protein content levels before and after the removal of the lipid phase. The protein mass and concentration of the supernatant and precipitate were determined via the Kjeldahl nitrogen procedure, using 6.38 as the conversion factor (IDF Standard 20B, 1993) [27]. The surface protein mass (Γ, %) was calculated using the following Equation (1):

Γ = (P_0_ − P_1_)/Mc × 100% (1)

where P_0_ is the total protein in the emulsion (g), P_1_ is the remaining protein after the removal of the floating cream (g), and M_C_ is the weight of the emulsion (g).

#### 2.3.7. Viscosity

Following a storage period of 4 °C for 24 h, the viscosity of the emulsions was measured using a viscometer (DV3T, Brookfield, Middleboro, MT, USA) coupled with an SC4-18 spindle at 5 rpm. The measurements were performed in triplicate.

### 2.4. Characterization of Aerated Emulsions

#### 2.4.1. Overrun

The overrun, or amount of air introduced into each aerated emulsion, was determined at 4 °C. According to Fredrick et al., 2013 [22], overrun can be calculated by comparing the mass of equal volumes of unwhipped and whipped emulsions, according to the following Equation (2):

Overrun = (m_1_ − m_2_)/m_2_ × 100% (2)

where m_1_ is the mass of the unwhipped emulsion, and m_2_ is the mass of the aerated emulsion, measured three times after each whipping.

#### 2.4.2. Firmness

At 4 °C, deformation puncture measurements were taken with a texture analyzer (TA500, Largo, FL, USA) equipped with an acrylic cylindrical probe. Puncture tests were performed on the surface of the aerated emulsions at a rate of 1 mm/s over 20 mm. The trigger threshold value for the start of each measurement was set at 0.01 N. The force required to reach this depth (20 mm) was defined as the firmness of the aerated emulsions. The sensitivity of the load cell of the texture analyzer was 1000 g. Each sample was measured in triplicate.

### 2.5. Data Analysis

SPSS Statistics V21.0 software (IBM Inc., Chicago, IL, USA) was used to perform a one-way analysis of variance (one-way ANOVA). A difference of *p* < 0.05 was regarded as statistically significant.

## 3. Results and Discussion

### 3.1. Effects of pH on the Stability and Aeration Characteristics of Dairy Emulsion

#### 3.1.1. Emulsion Stability

**Figure 1:**
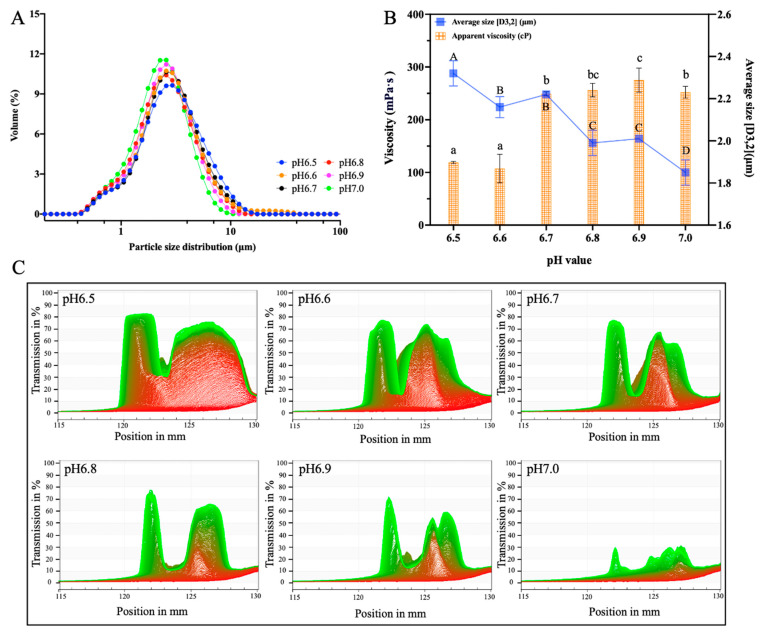
The effects of pH on (**A**) particle size distribution, (**B**) average size and viscosity, and (**C**) the scanning results of the dispersion analysis of the O/W emulsion. Different superscript letters ^a–c, A–D^ above the data points indicate significant differences (*p* > 0.05) among the various pH values. The initial transmission reading was presented by red lines while the final transmission curve was in green. The curves between them have gradual change in colors from red to green, corresponding to each measurement, to show the measurement process.

The distribution of particle sizes in the O/W emulsion with different pH levels was used to investigate fat aggregation [28]. As shown in Figure 1A, as the pH increased, the range of the peak area of the particle size distribution decreased and the peak gradually shifted towards smaller particle sizes. These results indicate that the increase in pH caused the particle size distribution range to become more centralized, which decreased the size of fat globules and made the emulsion more homogeneous and stable. In Figure 1B, the particle size of fat droplets showed a significant decrease from 2.32 ± 0.06 μm (pH 6.5) down to 1.99 ± 0.06 μm (pH 6.8), stabilizing at pH 6.9 (*p* > 0.05), then decreasing further to 1.85 ± 0.06 μm (*p* < 0.05) when pH was 7.0. The increase in pH improved the emulsification characteristics of the milk protein, which enhanced the better intercalation of milk protein in the O/W phases, resulting in a decrease in the fat globule droplet sizes.

The viscosity of the emulsions showed a tendency to increase as the pH increased (Figure 1B), rising significantly (*p* < 0.05) to 249.21 ± 3.86 CP at pH 6.7, but thereafter decreasing when the pH reached 7.0. At pH 6.9, the viscosity of the emulsion was highest at 275.21 ± 22.87 CP. As the pH decreased, a slight increase in ionic strength was evident, with the protein net charge reduced towards neutrality, the intermolecular electrostatic repulsive forces lessened, and non-ionic protein–protein attractive interactions favored; therefore, the viscosity increased [29].

Creaming is inevitable in almost all O/W emulsions and can be greatly enhanced under centrifugation by the difference in the mass density of the dispersed oil phase and the continuous aqueous phase [30]. Figure 1C displays the recorded evolution (from right to left) of time-dependent transmission profiles for the O/W emulsions at pH 6.5~7.0. As the pH decreased, the emulsion transmission area became larger, an obvious oil–water separation phenomenon appeared, and the O/W emulsion was more stable at pH 6.8~7.0. Thus, it was evident from the results that pH played an important role in the stability of the O/W emulsions. The stability was enhanced when the pH gradually increased away from the protein isoelectric point. The electrostatic repulsions of proteins gradually increased with the deviation of pH conditions from the isoelectric point [31,32,33], followed by an increase in the physical stability of the O/W emulsions.

#### 3.1.2. Aeration Characteristics

**Figure 2:**
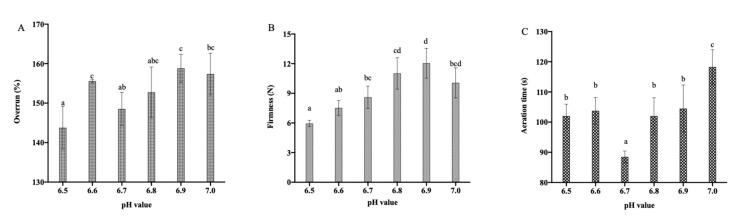
The effect of pH on overrun (**A**), foam firmness (**B**), and aeration time (**C**) of the aerated emulsion. Different superscript letters ^a–d^ above the data points indicate significant differences (*p* > 0.05) among the various pH values.

The aeration characteristics of the dairy emulsion under different pH are shown in Figure 2. The lowest overrun was 143.77 ± 5.42% at pH 6.5, while the highest overrun was 157.37 ± 5.29%% at pH 7.0. The foam firmness increased when the pH increased from 6.5 to 6.9 and was lowest at 5.95 ± 0.32 N at pH 6.5 and highest at 12.05 ± 1.51 N at pH 6.9. The whipping time tended to decrease at first and then increase in the pH range of 6.5~7.0, with the shortest whipping time noted at pH 6.7. When the pH was higher than 6.7, the whipping time increased with the increase in pH but showed no significant difference between pH 6.5 and 6.6 (*p* > 0.05).

Protein aggregation was maximized when the pH was 6.5 close to the protein isoelectric point [9]. As the pH deviated from the protein isoelectric point, the protein emulsification characteristic was enhanced, and the interfacial protein mass was reduced. The lower the interfacial protein mass and membrane strength, the more likely it was that fat crystal could puncture the fat globule membrane and the easier it became for the transition phase to occur. Therefore, the fat globule partially coalesced, and air bubbles could be effectively enveloped. Consequently, the whipping time was shorter, and the overrun and firmness were higher. Moreover, the emulsions at a higher pH were found to be vicious, leading to aeration resistance and extending the time it took to complete aeration.

Overall, better emulsion stability and aeration characteristics were achieved at a pH of 6.8 to 7.0. The mechanism whereby pH affects the stability and aeration characteristics of the dairy emulsion was subsequently investigated with the emulsion pH fixed at 6.8 and 7.0 together with CaCl_2_ solutions with different concentrations.

### 3.2. Ionic Strength with Multiple pH Values

**Figure 3:**
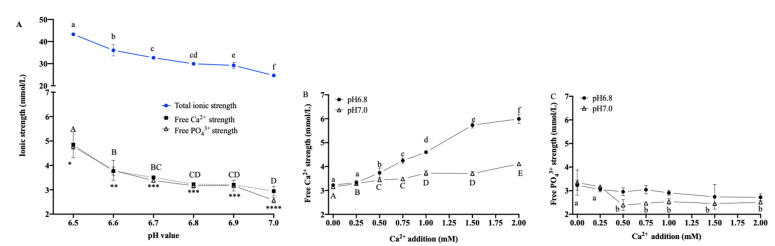
The effects of pH on (**A**) total ion strength and the effects of Ca^2+^ additions on (**B**) free Ca^2+^ strength and (**C**) PO_4_^3−^ strength in O/W emulsions. Different superscript letters ^a–f, A–E^ above the data points and *, **, ***, **** indicate significant differences (*p* > 0.05) among the various pH values and Ca^2+^ additions, respectively.

In Figure 3, the total ionic strength in the emulsions showed a significant decrease as the pH increased, from 43.28 ± 0.44 mM at pH 6.5 to 24.67 ± 0.65 mM at pH 7.0 (*p* < 0.05), which indicated that an increase in pH decreases the total ionic strength in O/W emulsion. It has been reported that the negative monovalent sodium chloride symporter (Na^+^/Cl^−^) has no significant effect on the stability of the O/W emulsions [4]. It is reasonable to conclude that it was mainly Ca^2+^ and phosphate (PO_4_^3−^) that affected the change in total ionic strength. The free Ca^2+^ and PO_4_^3−^ in the emulsions decreased significantly (*p* < 0.05) when the pH increased away from the protein isoelectric point. Free Ca^2+^ decreased from 4.85 ± 0.54 mg/L at pH 6.5 to 2.94 ± 0.20 mg/L at pH 7.0, and free PO_4_^3−^ decreased from 4.78 ± 0.12 mg/L at pH 6.5 to 2.57 ± 0.11 mg/L at pH 7.0 (*p* < 0.05). There were no significant differences in either of these free ions in the emulsions at pH 6.7 to 6.9. (*p* > 0.05).

With the increase in the additions of Ca^2+^, the free Ca^2+^ strength in the emulsion showed an increasing trend. The free Ca^2+^ strength in the control sample (without the addition of Ca^2+^) was 3.23 ± 0.06 mM and 3.15 ± 0.03 mM at pH 6.8 and pH 7.0, respectively, and continued to show significant increases to 5.99 ± 0.20 mM (pH 6.8) and 4.11 ± 0.02 mM (pH 7.0) when the Ca^2+^ addition was up to 2.00 mM. The free PO_4_^3−^ strength in the control sample was 3.23 ± 0.21 mM and 3.34 ± 0.54 mM at pH 6.8 and pH 7.0, respectively, and continued to show significant decreases to 2.72 ± 0.14 mM and 2.39 ± 0.23 mM when the Ca^2+^ additions were 2.00 mM and 0.50 mM, respectively. Thereafter, the free PO_4_^3−^ strength remained stable despite increased additions of Ca^2+^.

A dynamic equilibrium between the soluble free Ca^2+^ and PO_4_^3−^ content and insoluble colloids, such as CCP, was evident in the emulsion system, and the following reactions occurred as Ca^2+^ + H_2_PO_4_^−^ → CaHPO_4_ + H^+^. At a lower pH of 6.8, compared with pH 7.0, there was more H^+^ in the emulsion, so the degree of a positive shift in the equilibrium was lower. When Ca^2+^ was added to the emulsion, the increase in free Ca^2+^ strength was significant, but the free PO_4_^3−^ decreased slightly.

### 3.3. Effects of Ionic Strength on the Stability and Aeration Characteristics of Dairy Emulsion

#### 3.3.1. Zeta Potential

**Figure 4:**
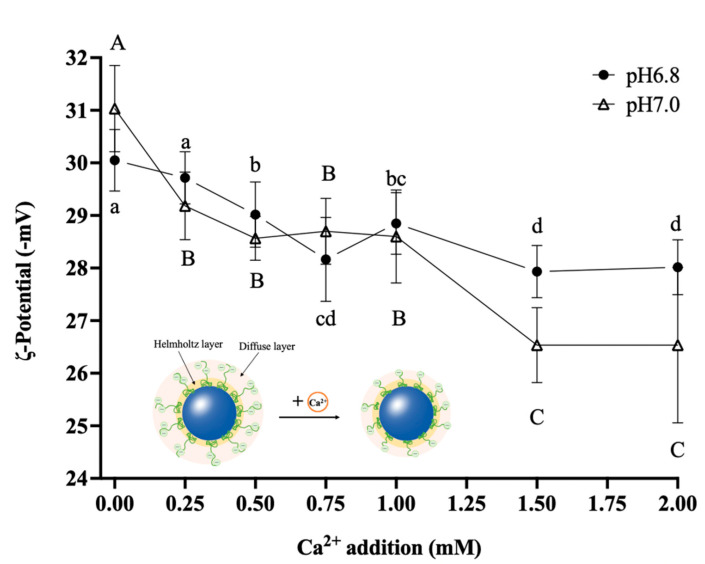
Effects of Ca^2+^ additions on the zeta (ζ)-potential of O/W emulsions. Different superscript letters ^a–d, A–C^ above the data points indicate significant differences (*p* > 0.05) among the various Ca^2+^ additions.

The zeta (ζ) potential accurately reflects the intensity of electrostatic interactions between emulsion droplets [32]. As shown in Figure 4, at pH 6.8, with the addition of 0.50 mM Ca^2+^, the absolute value of the ζ-potential decreased significantly from 30.05 ± 0.59 mV (without the additional Ca^2+^) to 29.02 ± 0.62 mV (*p* < 0.05) and continued to decrease thereafter until the Ca^2+^ addition reached 1.50 and 2.00 mM when the absolute value of ζ-potential was the lowest. At pH 7.0, the addition of 0.25 mM Ca^2+^ led to a significant decrease in the ζ-potential (*p* < 0.05), while the Ca^2+^ addition of 0.25~1.00 mM had no significant effect on the ζ-potential (*p* > 0.05). The absolute value of the ζ-potential decreased significantly to 27.93 ± 0.59 mV (*p* < 0.05) when Ca^2+^ was added at 1.50 mM and was not significantly different from that after the 2.00 mM Ca^2+^ addition.

In this study, the absolute ζ-potential values of all the samples presented a declining trend as the Ca^2+^ additions increased, indicating that the increasing Ca^2+^ reduced the negative charge on the surface of the emulsion protein. The electrostatic repulsion between Ca^2+^ and the original equilibrium ions in the diffusion layer squeezed some of the equilibrium ions into the adsorption layer (Figure 4), which reduced the thickness of the diffusion layer and lowered the absolute value of the ζ-potential. On the one hand, because the thickness of the diffusion layer was reduced, the distance between particles was shortened, the gravitational force was increased, the spatial potential resistance was reduced, and the interparticle aggregation was rapid, resulting in a loss of spatial stability in the emulsion. On the other hand, the phosphate group on casein could quickly bind with Ca^2+^, thereby promoting the interaction of hydrophobic regions, leading to the reduction of the charge density carried by the protein surface and the weakening of the electrostatic mutual repulsion between proteins, and causing the emulsion to lose electrostatic stability.

#### 3.3.2. Interfacial Proteins and Particle Size

**Figure 5:**
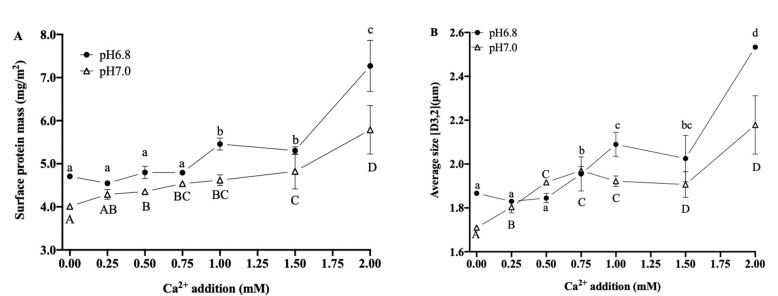
Effects of Ca^2+^ additions on interface protein mass (**A**) and particle size (**B**) of O/W emulsions. Different superscript letters ^a–c, A–D^ above the data points indicate significant differences (*p* > 0.05) among the various Ca^2+^ additions.

The surface protein content was calculated according to Equation (1) to estimate the proteins adhering to the interface of the fat globules. As shown in Figure 5, at pH 6.8 and pH 7.0, the additions of Ca^2+^ increased the interfacial protein mass of the fat globules, and the emulsions at pH 6.8 had an overall higher interfacial protein mass than those at pH 7.0. When the pH was 6.8, there was no significant difference in the interfacial protein mass after the addition of 0~0.75 mM Ca^2+^. However, when the Ca^2+^ addition was increased to 1.00 mM, the interfacial protein mass increased significantly to 5.46 ± 0.14 mg/m^2^ (*p* < 0.05), which was similar to that after the 1.50 mM addition (*p* > 0.05), while the further addition of Ca^2+^ to 2.00 mM led to a significant increase in the interfacial protein mass, to 7.27 ± 0.60 mg/m^2^. At pH 7.0, the interfacial protein mass showed a slow increase at a Ca^2+^ addition of less than 1.50 mM, from 4.01 ± 0.05 mg/m^2^ (without the addition of Ca^2+^) to 4.82 ± 0.41 mg/m^2^, but increased significantly (*p* < 0.05) to 5.79 ± 0.56 mg/m^2^ when the Ca^2+^ addition reached 2.00 mM.

Particle size analysis was performed to assess the effects of Ca^2+^ additions on the physical stability of O/W emulsions. As shown in Figure 5, the average particle sizes in the emulsion at pH 6.8 and pH 7.0 showed an overall increasing trend (*p* < 0.05) as the addition of Ca^2+^ increased. At pH 6.8, the additions of Ca^2+^ were 0.75 and 1.00 mM; the particle size increased from 1.87 ± 0.01 μm (without additional Ca^2+^) to 1.96 ± 0.08 μm and 2.09 ± 0.05 μm, respectively (*p* < 0.05), and reached a maximum value of 2.53 ± 0.01 μm at 2.00 mM of Ca^2+^ addition, indicating the increased degree of bridging flocculation of fat globules and leading to damage in the emulsion structure. At pH 7.0, the particle sizes increased significantly at 0.25 and 0.50 mM of Ca^2+^ addition.

In this work, the binding of Ca^2+^ and protein may promote mutual aggregation and polymerization between the protein molecules and reduce their electrostatic repulsion, leading to increased interfacial protein mass, reducing protein emulsification characteristics, changing protein structure, and affecting protein adsorption to the O/W interface. The proteins could not unfold and rearrange effectively at this point and, thus, did not immediately cover the surface of the newly formed fat globules during homogenization, while small fat globules had high interfacial free energy, which, in turn, coalesced into larger fat globules. In addition, Ca^2+^ may also have caused the secondary adsorption of liquid-phase proteins and fat globule surface proteins, resulting in the thickening of fat globule membranes and increasing particle size.

#### 3.3.3. O/W Emulsion Stability

**Figure 6:**
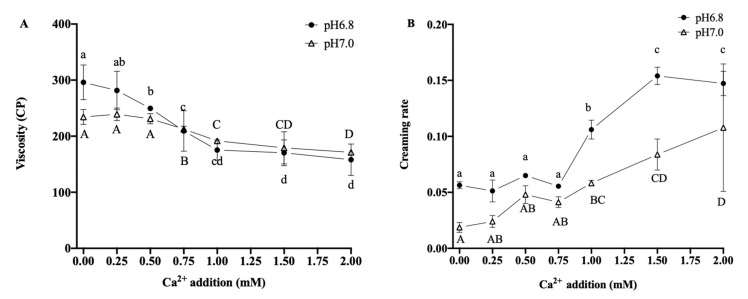
The effect of Ca^2+^ additions on the viscosity (**A**) and creaming rate (**B**) of the O/W emulsion. Different superscript letters ^a–d, A–D^ above the data points indicate significant differences (*p* > 0.05) among the various Ca^2+^ additions.

The viscosity decreased (*p* < 0.05) with increasing additions of Ca^2+^, as shown in Figure 6. At pH 6.8, the viscosity was 175.32 ± 18.03 CP when the addition of Ca^2+^ was 1.00 mM and remained stable when the Ca^2+^ addition was increased to 2.00 mM. At pH 7.0, there was no significant difference in viscosity after the addition of Ca^2+^ at 0~0.50 mM. However, the viscosity significantly decreased (*p* < 0.05) when the Ca^2+^ additions were increased to 0.75 and 1.00 mM and were at their lowest value of 171.21 ± 3.42 CP after the addition of 2.00 mM of Ca^2+^.

At pH 6.8 and pH 7.0, the creaming rate of the emulsions increased when the additions of Ca^2+^ were more than 0.75 mM, indicating that the emulsion was gradually destabilized by the increasing Ca^2+^. The creaming rates at pH 7.0 were lower than those at pH 6.8, indicating that the higher pH value enhanced the stability of the emulsion. However, when Ca^2+^ addition was 1.50 mM, at pH 6.8, the creaming rate increased to 0.15 ± 0.01 and was not significantly different after the addition increased to 2.00 mM. Still, at pH 7.0, the creaming rate was the highest (0.11 ± 0.02), and hence, the emulsion was least stable, when the additional Ca^2+^ was 2.00 mM.

With the increased Ca^2+^ addition, the interfacial protein mass increased while the protein in the aqueous phase was reduced, weakening the cross-linking between proteins and decreasing the viscosity. In addition, the average particle size of fat globules increased, while the number of fat globules decreased at the same fat content, thereby increasing the spacing between fat globules and decreasing the potential spatial resistance so that fat globules could move more easily than before. Moreover, the lower absolute ζ-potential value of the emulsion demonstrated that the electrostatic repulsion between the fat globules was greatly weakened, and the physical stability of the emulsions became unable to maintain [34].

#### 3.3.4. Aeration Characteristics

Table 1 lists the aeration characteristics of the dairy emulsions at pH 6.8 and pH 7.0. The optimum whipping time can be used to evaluate the partial coalescence of dairy cream. In whipping cream, a shorter aeration time indicates an increase in surface-mediated partial coalescence at an air or water interface [35]. In this study, the aeration time was reduced with the increase in the Ca^2+^ additions and was shorter at pH 6.8 than at pH 7.0 with the same Ca^2+^ additions. At pH 6.8, compared with the aerated emulsion without the additional Ca^2+^, aeration time was significantly reduced (*p* < 0.05) to 102.5 ± 5.3 s by the addition of 0.50 mM of Ca^2+^. The shortest aeration time was 92.3 ± 4.2 s when the Ca^2+^ addition was 2.00 mM.

Overrun is an indication of foaming capacity and fat globule network formation and is, thus, a crucial indicator of the extent of whipping [36]. The overruns of the aerated emulsions did not vary considerably at pH 6.8 or 7.0 when the increasing Ca^2+^ additions were lower than 0.75 mM. The overruns were significantly reduced at pH 7.0 with a Ca^2+^ addition of more than 0.75 mM, and the lowest overrun was 149.98 ± 2.78% with a Ca^2+^ addition of 2.00 mM. Foam firmness, another important parameter of aerated emulsions, is influenced by several factors, including interfacial protein mas, overrun, and fat globule coalescence [37]. There was no significant effect on the foam firmness at pH 6.8 and Ca^2+^ additions of 0~0.50 mM (*p* > 0.05). However, when the Ca^2+^ addition was increased to 0.75 mM, foam firmness decreased significantly (*p* < 0.05) but remained stable until the Ca^2+^ addition was increased to 2.00 mM.

### 3.4. Effects of Free Ca^2+^ Strength on Emulsion Stability and Aeration Characteristics

**Figure 7:**
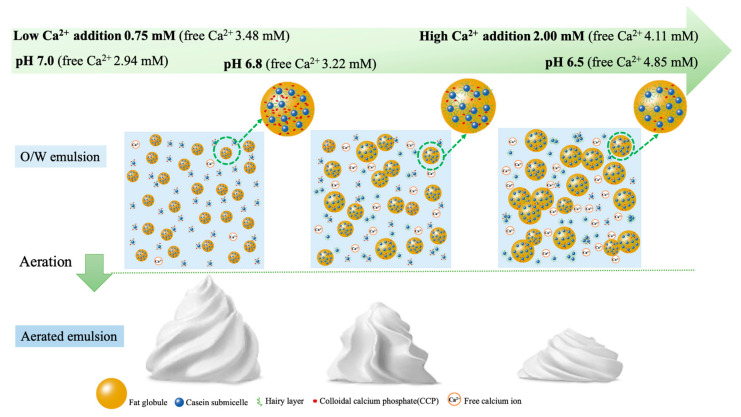
The schematic of the effect of pH and Ca^2+^ addition (free Ca^2+^ strength) on the casein micelle and the stability of O/W, aerated emulsion.

The surface-active behavior and hydrodynamic interactions between fat globules in emulsions can be altered by pH changes. The net charge of casein micelles was found to decrease as the pH decreased from 6.7 in milk to 4.6 at its isoelectric point [38,39,40]. At higher pH (7.0), Ca^2+^ could bind more strongly to phosphate, thereby avoiding binding to the phosphoserine site of casein and reducing the instability of the emulsion. Our results indicated that a moderate concentration of Ca^2+^ (free Ca^2+^ strength was around 3.25 ± 0.25 mM) at pH 6.8~7.0 could inhibit coalescence, thereby greatly enhancing emulsion stability and aeration characteristics. As shown in Figure 7, a lower pH and increased additions of Ca^2+^ led to higher free Ca^2+^ strength (free Ca^2+^ strength 4.11~4.85 mM), resulting in the progressive protonation of organic and inorganic phosphates and causing the progressive dissolution of CCP, which is the most important factor to decrease the stability of an O/W emulsion. Thereafter, the size of the fat globules became larger, leading to flocculation and coalescence, and the instability became increasingly severe with the decline in pH.

## 4. Conclusions

This study demonstrated the effects of pH and ionic strength on the stability and aeration characteristics of dairy emulsions, such as whipping/whipped cream, which could be used in coffee, egg tart, ice cream, and chocolate. Results showed that either pH changes or CaCl_2_ addition primarily influenced the emulsion properties by influencing free Ca^2+^ strength. When the free Ca^2+^ strength was around 3.25 ± 0.25 mM, the ζ-potential increased, and the fat globules were less prone to aggregate, which improved emulsion stability. The aeration characteristics, such as overrun, and foam firmness also increased. The findings of this study provide guidance and theoretical support for improving the stability of aerated emulsions from the perspective of ionic strength control.

## Figures and Tables

**Table 1:** The effect of Ca^2+^ additions on the aeration characteristics of the dairy emulsion.

pH	Aeration Characteristics	Ca^2+^ addition (mM)						
		0	0.25	0.50	0.75	1.00	1.50	2.00
6.8	Aeration time/s	116.00 ± 11.37 ^a^	114.25 ± 2.22 ^ab^	102.5 ± 5.32 ^c^	106.25 ± 4.72 ^bc^	105.25 ± 7.14 ^bc^	98.75 ± 1.50 ^cd^	92.33 ± 4.16 ^d^
	Overrun/%	158.44 ± 2.26 ^ab^	159.99 ± 3.24 ^a^	159.43 ± 0.64 ^a^	155.17 ± 0.94 ^ab^	154.41 ± 5.12 ^ab^	153.07 ± 2.88 ^b^	153.31 ± 2.48 ^b^
	Foam firmness/N	10.02 ± 1.50 ^a^	11.00 ± 1.30 ^a^	9.77 ± 1.39 ^a^	5.89 ± 2.02 ^b^	6.23 ± 1.38 ^b^	6.10 ± 1.25 ^b^	5.65 ± 2.45 ^b^
7	Aeration time/s	146.25 ± 4.99 ^A^	147.67 ± 11.24 ^A^	121.25 ± 2.87 ^B^	121.75 ± 9.22 ^B^	108.5 ± 1.29 ^C^	106.25 ± 6.39 ^C^	101.00 ± 5.944 ^C^
	Overrun/%	177.08 ± 2.32 ^A^	175.89 ± 2.42 ^A^	175.116 ± 0.79 ^A^	173.73 ± 3.56 ^A^	166.63 ± 2.49 ^B^	159.15 ± 5.85 ^B^	149.98 ± 2.78 ^C^
	Foam firmness /N	10.21 ± 0.88 ^A^	8.72 ± 2.68 ^A^	8.99 ± 2.77 ^A^	9.58 ± 1.36 ^A^	8.51 ± 1.30 ^A^	8.56 ± 2.85 ^A^	6.34 ± 0.29 ^A^

Different letters ^a–d, A–C^ within the same row are significantly different (*p* < 0.05). Mean ± SD, n = 3.

## Data Availability

Data is contained within the article.
